# Association Between Serum Ferritin and the Duration of Type 2 Diabetes Mellitus in a Tertiary Care Hospital in Chennai

**DOI:** 10.7759/cureus.53117

**Published:** 2024-01-28

**Authors:** Sankar Arumugam, Anbalagan Suyambulingam

**Affiliations:** 1 General Medicine, Sree Balaji Medical College and Hospital, Chennai, IND

**Keywords:** fasting blood sugar, serum ferritin, oxidative stress, insulin resistance, diabetes mellitus

## Abstract

Background: Hyperinsulinemia has been linked to increased ferritin production and iron absorption in type 2 diabetes mellitus, ultimately leading to increased iron storage. Glucose intolerance is intimately linked to this issue. Increased oxidative stress from iron decreases insulin's ability to be taken into cells and used for energy. Researchers suggest that increased iron levels in the body play a role in the emergence of insulin resistance, glucose intolerance, and vascular repercussions associated with diabetes.

Objective: The aim of this study is to assess the levels of serum ferritin and fasting plasma glucose in both diabetic and nondiabetic individuals while establishing a relationship between the two. Exploring the connection between serum ferritin levels and the duration of diabetes mellitus in individuals diagnosed with diabetes is our objective.

Methodology: In this study, 80 men diagnosed with type 2 diabetes mellitus were included, and they were compared with 70 male volunteers who were in good health. We took blood samples while the subjects fasted, and we analyzed the plasma glucose and serum ferritin levels.

Results: In the diabetic group, there were notably higher levels of serum ferritin and fasting plasma glucose compared to the nondiabetic subjects. Furthermore, a correlation was observed between the duration of diabetes among participants with diabetes and elevated serum ferritin levels.

Conclusion: The findings suggest that low-grade inflammation and increased body iron stores are positively related to hyperglycemia in type 2 diabetes mellitus.

## Introduction

Diabetes mellitus is a serious health condition that progressively impacts various tissue and organ systems, significantly affecting the quality of life. The development of a vicious cycle involving increased insulin resistance and subsequent complications is a key characteristic of this disease. Increased serum ferritin and body iron reserves have emerged as possible contributions among the many risk variables and causes that have been discovered. The purpose of this investigation is to examine how elevated blood ferritin levels correlate with how long a person has had diabetes.

Due to its redox nature, iron plays a crucial part in the pathophysiology of diabetes by being oxidized and reduced in a continuous cycle, which in turn generates reactive oxygen species via the Haber-Weiss reaction. Four pathways have been proposed by which iron affects diabetes: (a) insulin shortage since pancreatic islets are prone to oxidative damage from free radicals; (b) insulin resistance; (c) hepatic dysfunction; and (d) gathering of iron in the interstitial cells of the pancreas leading to the deposition of collagen and impaired microcirculation [1].

Research findings indicate a direct and independent correlation between increased iron reserves in the body and the occurrence of metabolic disorders, raised fasting glucose levels, and dyslipidemia [2,3]. This is because free radical damage caused by elevated iron storage increases the risk of diabetes complications. Rajpathak et al. (2009) and De Sanctis et al. (2007) are only two of the many research groups that have established a correlation between high iron reserves and an increased chance of creating type 2 diabetes mellitus [4,5]. People with iron overload illnesses, such as hemochromatosis, or those who need regular transfusions, such as those with thalassemia, are also at an increased risk of developing diabetes [5].

There is epidemiological data to show that consuming more heme, which is abundant in animal products like meat, increases one's chance of developing diabetes [6]. Oxidative stress may cause free radicals to form, and these radicals can damage pancreatic beta cells, resulting in less insulin being produced and secreted [7]. Reduced levels of cancer prevention agent catalysts, for example, superoxide dismutase, catalase, and glutathione peroxidase, make pancreatic islet cells more susceptible to oxidative injury [8]. In addition, studies have shown that phlebotomy, a process used to remove extra iron from the body, may boost insulin sensitivity [9]. In response to inflammatory stress, serum ferritin levels rise, which indicates an increase in iron reserves and may be used as a biomarker [10].

In light of these observations, the current study seeks to determine whether there is a correlation between raised blood ferritin levels and the perseverance of diabetes. This study has the potential to enhance diabetes treatment and preventive measures, as well as dietary advice, by increasing our knowledge of the role that iron stores play in the improvement of diabetes and its consequences.

## Materials and methods

The Department of General Medicine at a tertiary care hospital (Sree Balaji Medical College, Chromepet, Chennai, Tamil Nadu) provided the setting for this cross-sectional research. Ethical considerations were considered, and permission to conduct the research was granted by the appropriate institutional review board. From April 2023 to June 2023, the research was conducted. The samples, totaling 150, were split evenly between the diabetics (n = 80) and the controls (n = 70).

Group members with diabetes were all men with simple type 2 diabetes, with ages ranging from 25 to 70. Male participants of the same age who had fasting blood glucose levels below 80 mg/dl served as the control group. Women were not included since menstruation affects serum ferritin levels; therefore, they could not be the study's subjects. Serum ferritin levels tested during the menstrual phase tend to exhibit lower values compared to the luteal and late luteal phases [11].

People with complicating comorbidities, including type 1 diabetes, hemochromatosis, constant liquor addiction, ongoing provocative circumstances (for example, foundational lupus erythematosus, hepatitis), those undergoing frequent blood transfusions, individuals experiencing iron deficiency anemia, recent episodes of blood loss, hemorrhoids causing bleeding, recent significant surgeries, diabetic foot ulcers, hypothyroidism, cardiovascular disorders, diabetic nephropathy, and anemia stemming from any cause, were also excluded from the study.

Blood samples (4.0 ml) were taken from each participant's vein while they were fasting and were handled with the utmost care and cleanliness. The enzymatic colorimetric methodology utilizing hexokinase and glucose 6-phosphate dehydrogenase was used to determine fasting plasma glucose levels. The normal range for fasting plasma glucose was defined as less than 100 mg/dl [12]. The Architect's chemiluminescent microparticle immunoassay approach was used to determine serum ferritin levels. Results between 21 and 274.66 ng/ml for serum ferritin were deemed normal.

We used IBM SPSS Statistics for Windows, Version 27 (Released 2020; IBM Corp., Armonk, New York, United States) to analyze the data. Fasting plasma glucose and serum ferritin levels were characterized by means and standard deviations (SDs) and other descriptive data. When comparing the two groups, these parameters were analyzed using independent sample t-tests. We used Pearson correlation coefficients to examine the linear relationship between serum ferritin and fasting plasma glucose and diabetes term.

## Results

Data suggests that fasting plasma glucose was 162.92 mg/dl and an SD of 42.9 mg/dl among diabetics and 88.96 mg/dl with a standard deviation of 12.1 mg/dl among healthy individuals. The researchers found a statistically significant distinction between the two groups, as indicated by a p-value below 0.001 (Table 1).

**Table 1:** Mean fasting plasma glucose (FPG) comparison. FPG values are expressed in mg/dl. The p-value indicates the significance of the difference between the two groups.

Group	N	Mean	Standard Deviation	Standard Error	p-value
Diabetics	80	162.91	42.976	4.805	0.000
Control	70	88.96	12.118	1.448	

In the diabetes group, serum ferritin was measured at 291.52 ng/ml and an SD of 93.12ng/ml on average compared to 83.23 ng/ml and an SD of 56.16 ng/ml in the control group. Significant (p = 0.001) differences in serum ferritin levels between the two groups were found (Table 2).

**Table 2:** Mean value of serum ferritin comparison. Serum ferritin levels are expressed in ng/dl. The p-value indicates the statistical significance of the difference between the two groups.

Group	N	Mean	Standard Deviation	Standard Error	p-value
Diabetics	80	219.3875	93.87596	10.49565	0.000
Control	70	83.4686	56.01362	6.69491	

Concentrations of fasting plasma glucose were positively correlated with serum ferritin. The estimation of the correlation coefficient (r) yielded a value of 0.810, showing a quite certain connection between the two factors. Given that the p-an incentive for this connection coefficient is below 0.001, it is evident that a robust relationship exists between the two variables. There appears to be a positive relationship between fasting plasma glucose levels and serum ferritin levels. Besides, a noticeable positive connection was noted between serum ferritin levels and the term diabetes (r = 0.2185, p = 0.035) (Table 3).

**Table 3:** Serum ferritin with fasting plasma glucose (FPG) and duration of diabetes: a linear correlation. FPG refers to fasting plasma glucose levels. The correlation coefficients (R) and corresponding p-values indicate the strength and statistical significance of the linear correlations between serum ferritin and FPG and the duration of diabetes.

Group	FPG	Duration of Diabetes
Serum Ferritin	R = 0.810, p < 0.001	R = 0.185, p = 0.035

Fasting plasma glucose was demonstrated to be essentially corresponded with serum ferritin levels. Estimates of the correlation coefficient (r) between the two variables suggest a strong positive relationship. The association between these two factors is significant, with a p-value of less than 0.001. Based on these findings, it seems that an increase in serum ferritin levels is accompanied by an equivalent rise in fasting plasma glucose (Table 3).

These findings indicate that raised serum ferritin levels in diabetics are exceptionally connected with rising fasting plasma glucose levels. What's more, a positive association between serum ferritin levels and diabetes duration was discovered, with a value of r equal to 0.185 (p = 0.035). According to these results, serum ferritin rises in tandem with the length of time spent living with diabetes (Table 3).

As seen above, elevated serum ferritin levels are linked to both higher fasting plasma glucose levels and longer durations of diabetes. Evidence from this study lends credence to the idea that higher blood ferritin levels contribute to the development of diabetes.

## Discussion

The interplay between metabolism and immunity is crucial for maintaining overall health. However, under metabolic stress conditions, this relationship can become detrimental. Inflammation, which activates catabolic pathways and suppresses anabolic pathways like insulin signaling, can lead to insulin resistance [13]. Consistent with prior research, we found higher mean serum ferritin levels in the diabetic gathering, lending credence to the hypothesis that inflammation, insulin resistance, and the prevalence of type 2 diabetes are interconnected pathways [14]. In addition to being an inflammatory marker [15], ferritin is a useful indication of iron storage in the body [16]. It serves a crucial function in maintaining a steady level of iron inside the cell and regulating its release.

Hyperinsulinemia and insulin resistance occur because iron is a strong favorable oxidant that increments cell oxidative pressure [17]. This in turn reduces insulin internalization and activity. Due to glycation, ferritin loses its iron-binding ability and free iron levels rise. This, in turn, increases ferritin synthesis [18]. In the complex course of cutting-edge glycation final result union, people with diabetes create receptive oxygen species through metal-catalyzed responses. These responsive oxygen species slow down insulin receptor movement and block the movement of the glucose carrier GLUT4 to the plasma layer. As a result, insulin signaling is disrupted at various levels [19].

Furthermore, serum ferritin levels have been linked to a higher likelihood of developing ischemic heart disease [20]. As a result, learning more about iron overload status in people with diabetes may provide light on oxidative stress, insulin resistance, and the likelihood of developing diabetic vascular problems. There seems to be a correlation between the severity of neurologic and vascular problems, the length of diabetes, serum ferritin levels, body iron stores, and oxidative pressure.

The study's cross-sectional design limits its ability to establish causation and temporal relationships, while its exclusive focus on men with type 2 diabetes and male controls, along with a lack of participant information, hinders generalizability. Using serum ferritin as an indicator without accounting for potential influences like inflammation adds complexity.

Our findings emphasize the need to evaluate iron overload in diabetes for its insight into insulin resistance, oxidative stress, and the progression of diabetic vascular problems. Managing and preventing diabetes-related problems requires therapies that target iron metabolism and oxidative stress, as shown here.

## Conclusions

Our research results reveal that persons with diabetes had higher blood ferritin levels, which were positively linked with hyperglycemia and the duration of diabetes. Insulin resistance is linked to ferritin levels because they indicate iron excess. To measure body iron storage and determine the risk of developing diabetes, regular evaluation for serum ferritin levels ought to be investigated in individuals with weakened glucose resistance.

Divalent metal transporter 1 (DMT1), ferroportin, and metal tolerance protein 1 (MTP1), transporters responsible for the absorption of iron in the intestines and its entry into the bloodstream, and hephaestin, which catalyzes the oxidation of ferrous ions to ferric ions during this process, are key regulators of iron metabolism that should be investigated further to determine if they are altered in diabetes. Furthermore, there is much to be learned by comparing the diabetes rates of vegetarian and nonvegetarian communities.

To reduce body iron stores and oxidative stress, interventions such as blood donation or phlebotomy can be considered. Furthermore, it is advisable to significantly reduce the consumption of red meat and meat products in the diet.

In conclusion, our study highlights the importance of monitoring serum ferritin levels in individuals with hindered glucose resistance and emphasizes the need for interventions targeting iron metabolism and dietary choices to mitigate the risk of diabetes development and its associated complications. Future research in this area will provide a more comprehensive understanding of iron metabolism dysregulation in diabetes and pave the way for effective preventive and management strategies.
